# Biparametric MRI-based radiomics for differentiating clinically significant prostate cancer among prostate-specific antigen level of gray zone

**DOI:** 10.3389/fonc.2025.1615005

**Published:** 2025-08-27

**Authors:** Yugang Ji, Wei Liu, Houdong Liu, Jing Wen

**Affiliations:** ^1^ Department of Radiology, Yacheng First People's Hospital; Yacheng First Hospital Affiliated of Nanjing Medical College, Yancheng, China; ^2^ Department of Radiology, The People's Hospital of Tinghu District, Yancheng, China; ^3^ Department of Medical Imaging, Jiangsu Medical College, Yancheng, China

**Keywords:** bpMRI, prostate cancer, PI-RADS, radiomics, diagnostic performance

## Abstract

**Purpose:**

This study was intended to evaluate the performance of biparametric MRI (bpMRI) radiomics for detecting clinically significant prostate cancer (csPCa) in men with prostate-specific antigen (PSA) of 4–10 ng/mL.

**Method:**

We retrospectively included 287 patients with PSA levels of 4–10 ng/mL. Radiomics features were extracted from two MRI protocols of T2-weighted imaging (T2WI) and diffusion-weighted imaging (DWI, with b-values of 0, 1000, and 2000 s/mm²), and then selected with the least absolute shrinkage and selection operator (LASSO) regression method. The apparent diffusion coefficient (ADC) maps were calculated from these images and used for analysis. The radiomics signature (Radscore) based on the most useful radiomics features was calculated with the logistic regression method. MRI/US fusion targeted biopsy results were used as the reference standard. Diagnostic performance was decided using the area under the receiver operating characteristic (ROC) curve (AUC), and compared with Delong’s test. Finally, a model integrating radiomics features and Prostate Imaging Reporting and Data System (PI-RADS) was constructed.

**Results:**

A total of 15 T2WI radiomics features and 12 from DWI features were retained after selection with LASSO regression. On the test set, radiomics outperformed PI-RADS, with an AUC of 0.928 (95% CI 0.868–0.988) vs. 0.807 (95% CI 0.705–0.908; P=0.04). Additionally, the combined nomogram generated higher diagnostic accuracy (AUC 0.955, 95% CI 0.905–1.00), significantly outperforming both PI-RADS (P=0.002) and radiomics alone (P=0.02).

**Conclusion:**

bpMRI-based radiomics exhibited promising diagnostic accuracy for the detection of csPCa, significantly outperforming either PI-RADS or PSAD among patients with PSA of 4–10 ng/mL. Furthermore, the developed nomogram integrating radiomics and PI-RADS could further enhance diagnostic performance.

## Introduction

Prostate cancer represents a significant global health issue, affecting approximately 10 million men worldwide, with 7 million experiencing metastatic disease (1, 2). PSA plays an important role in early detection, management, and surveillance of patients with high risk of PCa (3, 4). In many developed countries, PSA level higher than 3-4.0 ng/mL was recommended prostate biopsy (5). Nevertheless, the elevation of PSA also can be caused by benign prostatic hyperplasia or prostatitis, thereby leading to high sensitivity but low specificity when using PSA as an independent predictor for PCa (5). Moreover, for patients with PSA levels of 4–10 ng/mL (often mentioned as the gray zone), the detection of csPCa is merely approximately 22%, resulting in many unnecessary biopsies or overtreatment for these populations (6, 7).

Currently, MRI plays a crucial role and is the primary imaging modality in the diagnosis, location, and management of PCa (8–10). PI-RADS, which is based on multiparametric MRI (mpMRI), has been widely applied in clinical practice. However, it is noteworthy that despite its high sensitivity, MRI exhibits lower specificity (11–13). Furthermore, mpMRI needs a long examination time to acquire DCE images, which prevents its application for some elderly people or claustrophobic patients (14). In recent years, radiomics has emerged as a promising technique and has been intensively studied in various diseases and preliminary studies have demonstrated the potential of radionics in PCa (15–17). Some studies employing radiomics in PCa used multiple MRI protocols including T2WI, dynamic contrast-enhanced (DCE) images, DWI, and apparent diffusion coefficient (ADC) maps (11, 18). Nevertheless, in recent years, many studies have shown that DCE has a marginal impact in evaluating lesions in the transitional zone (TZ) and can be omitted for those lesions in the peripheral zone (PZ) (19–21). As a result, bpMRI without DCE has been studied intensively, which can offer the benefits of shorter examination times and reduced costs, while maintaining similar performance to mpMRI (22–25). Therefore, in this study we intended to evaluate the accuracy of PSAD, PI-RADS v2.1, and bpMRI-based radiomics for the detection of csPCa among patients with PSA gray zone.

## Materials and methods

### Patient selection

**Figure 1:**
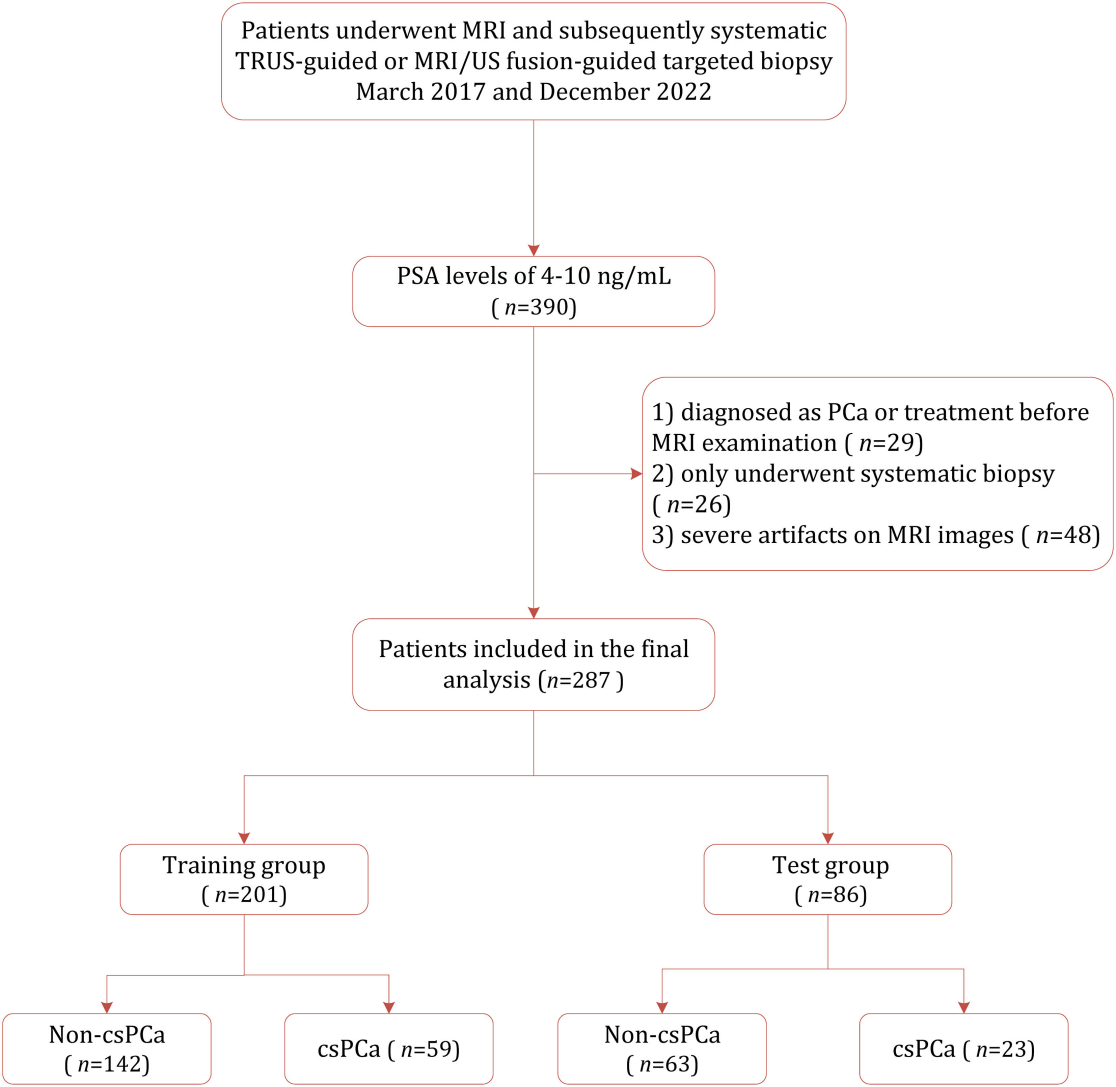
Study selection process for this systematic review and meta-analysis.

This retrospective study was approved by the institutional review board (IRB) of Yancheng First People’s Hospital, with the requirement for informed consent was waived. Between March 2017 and December 2022, 390 consecutive patients suspected of having PCa and with PSA between 4–10 ng/mL were identified from out institution. Of them, 103 were excluded because of the following reasons: (1) prior diagnosis or treatment of PCa (*n*=29); (2) underwent only systematic 10–12 core biopsy rather than MRI/US fusion targeted biopsy (*n*=26); and (3) presence of severe artifacts images on MRI (*n*=48). Consequently, 287 patients were included in the final study cohort, which was randomly divided into the training cohort (*n*=201) and the test cohort (*n*=86). **Figure 1** demonstrates the patient selection process.

### MRI acquisition and interpretation

All prostate MRI examinations were performed with a 3.0T scanner (MAGNETOM Skyra, Siemens AG). The bpMRI protocols included T1-weighted (T1WI), T2WI, and DWI. Detailed image acquisition parameters are provided in **Table 1**. All MRI images were independently interpreted by two genitourinary radiologists (with respective 2 and 4 years of experience), who were blinded to the final histopathology results and other clinical information.

**Table 1:** MRI parameters.

Parameter	T2WI (axial)	T2WI (sagittal)	DWI
Field of view (mm)	220×220	240×180	260×260
Acquisition matrix	276×240	104×125	104×125
Repetition time (ms)	3000	6000	6000
Echo time (ms)	100	77	77
Section thickness, no gaps (mm)	3.0	3.0	3.0
Acquisition time	4m 6s	3m 42s	3m 54s

DWI, diffusion weighted imaging; T2WI, T2-weighted imaging.

DWI performed with ***b*** values of 0, 100, 1000, 2000s/mm^2^.

### Prostate biopsy procedure

After the MRI examination, all lesions with suspicion of PCa underwent MRI/US targeted biopsy by an urologist with at least 16 years of experience. For suspected lesions, targeted biopsy cores were obtained from both axial and sagittal planes, with a minimum of two cores acquired per lesion. Prostate biopsy specimens were assessed by a genitourinary pathologist with more than 16 years of experience, and each lesion was assigned Gleason scores (GS), with the index tumor defined as the lesion with the highest GS score. In cases where more than one lesion shared the same highest GS, one with the highest tumor involvement percentage was selected as the index tumor. In this study, clinically significant PCa was defined as GS ≥7, and prostate volume was estimated using the ellipsoid formula. PSAD was calculated as the ratio of PSA to prostate volume (PSA/PV), and tumor size was obtained from T2WI.

### Feature extraction and radiomics analysis

Three-dimensional volumetric region of interest (ROIs) were manually delineated by 2 experienced radiologists (with 6 and 8 years of experience) on T2W images using 3D Slicer. Both radiologists were blinded to all clinical information, and all images were from MRI examination before targeted biopsy. These ROIs were then co-registered to the ADC maps to ensure spatial alignment prior to feature extraction. For co-registration, we employed a rigid registration approach, with mutual information as the similarity metric and nearest-neighbor interpolation to preserve ROI labels. This co-registration ensures that the features extracted from both modalities correspond to the same anatomical region, enabling meaningful multimodal radiomic analysis. Radiomic features were extracted from both T2W and DWI sequences with these 3D ROIs using PyRadiomics package (version 3.1.0). Unless otherwise specified, all feature extraction parameters followed the default settings in PyRadiomics. A total of 93 features were extracted from each original image sequence, including 18 first-order statistics, 24 Gray Level Co-occurrence Matrix (GLCM) features, 16 Gray Level Run Length Matrix (GLRLM) features, 16 Gray Level Size Zone Matrix (GLSZM) features, 14 Gray Level Dependence Matrix (GLDM) features, and 5 Neighboring Gray Tone Difference Matrix (NGTDM) features. Shape features were calculated in 3D based on the volumetric segmentation masks. No resampling was applied and the original voxel spacing of the images was retained. Gray-level discretization was conducted using the Fixed Bin Width (FBS) approach, with a default bin width of 25. For texture features, 13 directions in 3D were considered, with a pixel distance of 1. In addition to features extracted from the original images, we also extracted features from filtered images using PyRadiomics’ default image filters, including wavelet transforms and Laplacian of Gaussian (LoG) filters. For patients with more than one lesion, only the index lesion-either the one with the highest Gleason score or the largest size-was selected for evaluation. To ensure feature reliability, both inter- and intra-reader agreement for lesion segmentation and feature extraction were assessed using intraclass correlation coefficient (ICC), only those with ICCs ≥0.75 were retained for further analysis. The feature selection procedure was conducted using the LASSO regression method to select the most informative radiomics features. Radiomics signature (Radscore) was developed based on the top-ranked features, by using five-fold cross-validation on the training dataset to enhance model generalizability. We extracted 1040 radiomics features from T2WI (579 features) and DWI (561 features). After LASSO regression analysis, 27 robust non-zero coefficient features were retained to develop the final radiomics signature, including 15 features from T2WI and 12 from DWI. There was no significant difference in the Radscore between the training group and the test group (P=0.39). However, a significant difference in Radscore values was observed between csPCa and non-csPCa groups both in the training group (median 1.16 vs. -2.32, P<0.001) and in the test group (median 1.91 vs. -2.23, P<0.001).

### Statistical analysis

For continuous variables, were reported as mean ± standard deviation (normally distributed) or median and interquartile range (non-normally distributed), with comparisons performed with the independent t-test or Mann–Whitney U test, respectively. The Kolmogorov–Smirnov test was used to examine the normality of distribution for continuous variables. Categorical variables are presented as numbers and corresponding percentages, and compared with the chi-square (*χ^2^*) test. Given the imbalance between csPCa and non-csPCa cases, the Synthetic Minority Oversampling Technique (SMOTE) method was applied to solve this problem. The overall diagnostic performed was determined by AUC, and compared with DeLong’s test (26). Additionally, we calculated the sensitivity, specificity, positive likelihood ratio (LR+), and negative likelihood ratio (LR−). The best threshold for diagnostic performance was decided with the Youden index. The inter-reader agreements were assessed using Cohen’s kappa (κ) value which was interpreted as follows: <0.20, slight; 0.21-0.40, fair; 0.41-0.60, moderate; 0.61-0.80, substantial; and ≥0.81, almost perfect. Since multiple pairwise comparisons were performed among different models (Radiomics, PI-RADS, PSAD, and the combined model), P values from DeLong’s tests were adjusted using the Benjamini-Hochberg false discovery rate method to control for multiple testing (27). Statistical analyses were performed with R (version 4.3.2), with P values less than 0.05 indicated statistically significant. The net benefit of the combined model was assessed with the decision curve analysis (DCA), and the calibration curves were plotted to visually demonstrate the agreement between predicted probabilities and actual outcomes.

## Results

### Patient characteristics

**Table 2** demonstrates the characteristics of demographic and clinical for the study cohort. Among the 287 patients who underwent MRI/US fusion-targeted biopsy, 82 (28.57%) were diagnosed with csPCa, while the remaining 206 (71.43%) were either benign prostatic hyperplasia or non-clinically significant PCa. No statistically significant difference was found between the training cohort and the test cohort regarding age, PSA level, prostate volume, or PSAD, indicating good comparability between the two groups. However, the training set contained a higher proportion of lower GS values, while the test set includes relatively more high GS cases (P=0.04).

**Table 2:** Characteristics of patients.

Variable	Training (*n*=201)			Validation (*n*=86)		
	csPCa (*n*=59)	Non-csPCa (*n*=142)	*P*	csPCa (*n*=23)	Non-csPCa (*n*=63)	*P*
Age (Years, mean±SD)	71.57±8.75	68.33±7.53	0.01	70.28±8.54	68.06±7.77	0.03
PSA (ng/mL, median [IQR])	6.99 (5.58-8.00)	7.11 (5.60-8.52)	0.5	7.57 (6.10-9.01)	6.24 (5.25-7.99)	0.04
PV (ml, median [IQR])	38.08 (26.66-56.64)	58.02 (40.94-73.79)	<0.001	34.37 (25.40-46.18)	51.59 (40.40-70.76)	<0.001
PSAD (ng/mL/mL, median [IQR])	0.13 (0.16-0.23)	0.09 (0.12-0.16)	<0.001	0.15/0.18-0.31)	0.09 (0.11-0.17)	<0.001
Gleason score						
≤3+3	142			63		
3+4	30			9		
4+3	15			4		
4+4	5			8		
>4+4	9			2		
PI-RADS 2.1						
2	74			34		
3	54			25		
4	49			18		
5	24			9		

csPCa, clinically significant prostate cancer; IQR, interquartile range; PI-RADS, Prostate Imaging Reporting and Data System, version 2.1; PSA, prostate-specific antigen; PSAD, prostate-specific antigen density; PV, prostate volume; SD, standard deviation.

### Diagnostic performance of using PSAD and PI-RADS

For PSAD, the calculated AUC for the training cohort was 0.693 (95% CI 0.613–0.774), with an optimal threshold of 0.125 ng/mL/mL, where the sensitivity and specificity were 44.8% (95% CI 31.7%-58.5%) and 85.2% (95% CI 78.3%-90.6%), respectively. In comparison, the PI-RADS v2.1 achieved an AUC of 0.813 (95% CI 0.752–0.873), which outperformed PSAD (P=0.007). At a cutoff score of ≥3, PI-RADS achieved sensitivity of 94.8% (95% CI 85.6%-98.9%) and specificity of 46.5% (95% CI 38.1%-55.0%), respectively. A moderate inter-reader agreement was observed between the two radiologists, with a Cohen’s kappa value of 0.55 (95% CI: 0.52–0.60).

### Diagnostic performance of BpMRI radiomics

**Figure 2:**
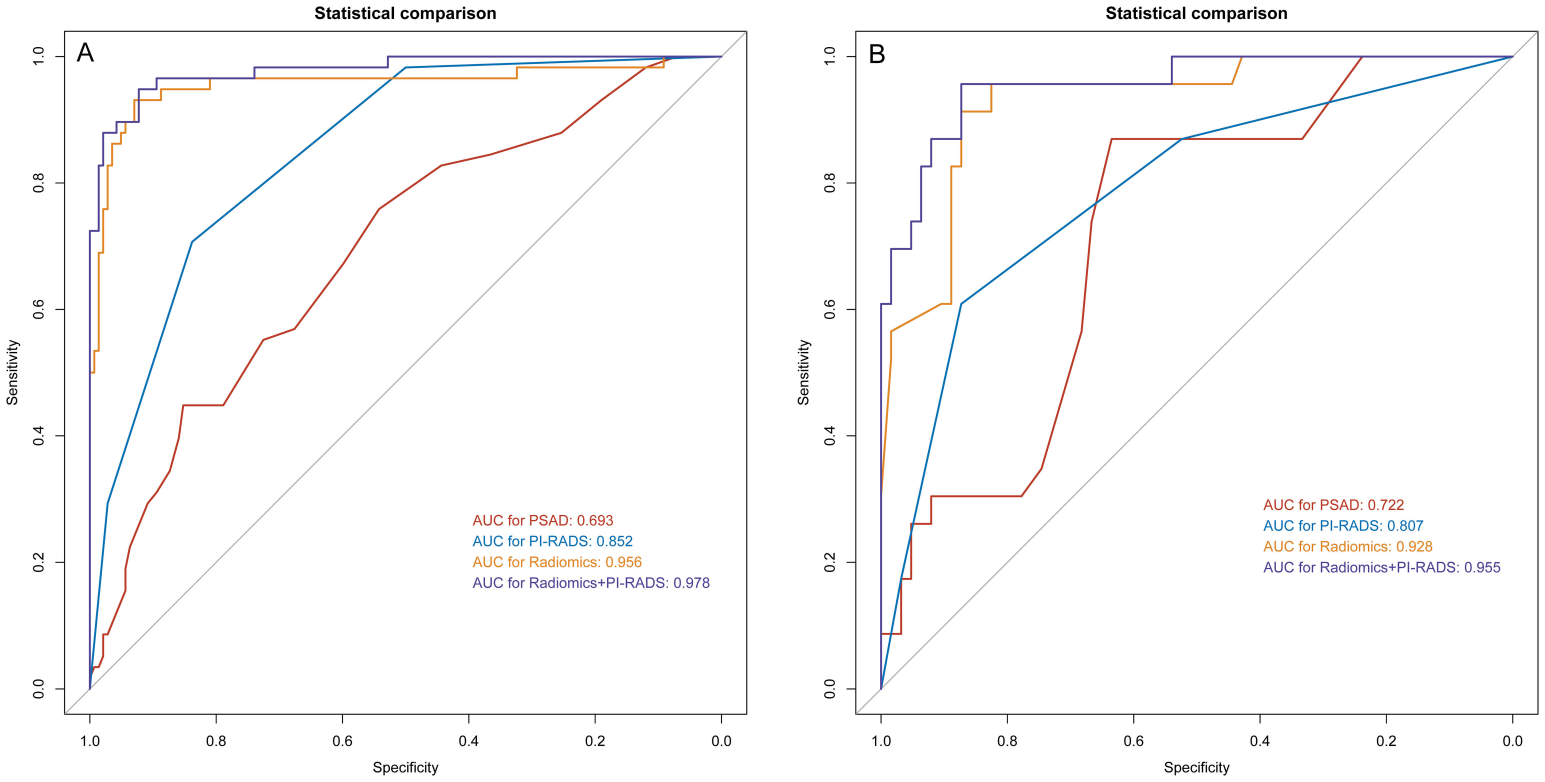
ROC analysis for PSAD, PI-RADS, radiomics, and the combination of PI-RADS and radiomics for prediction of clinically significant prostate cancer. **(A)** training group; **(B)** validation group. PI-RADS, Prostate Imaging Reporting and Data System, version 2.1; PSAD, prostate-specific antigen density.

**Figure 3:**
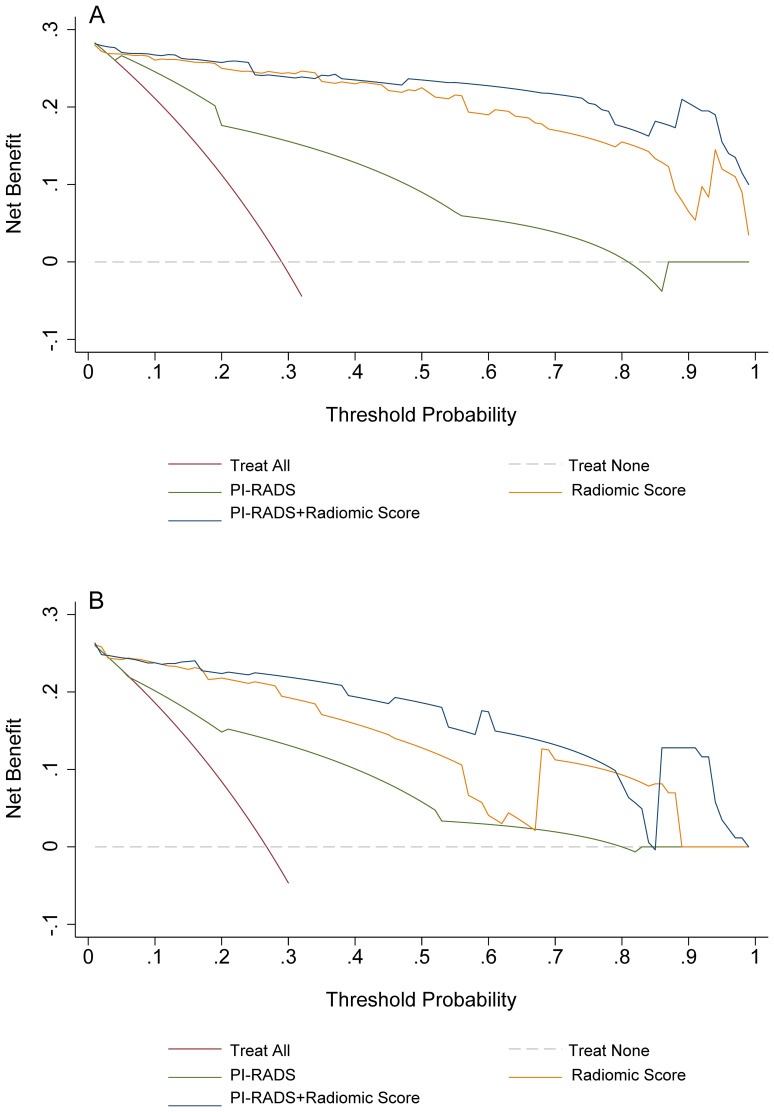
Decision curves analyses for PI-RADS, radiomics, and the combination of PI-RADS and radiomics for prediction of clinically significant prostate cancer. **(A)** training group; **(B)** validation group. PI-RADS, Prostate Imaging Reporting and Data System, version 2.1.

**Figure 4:**
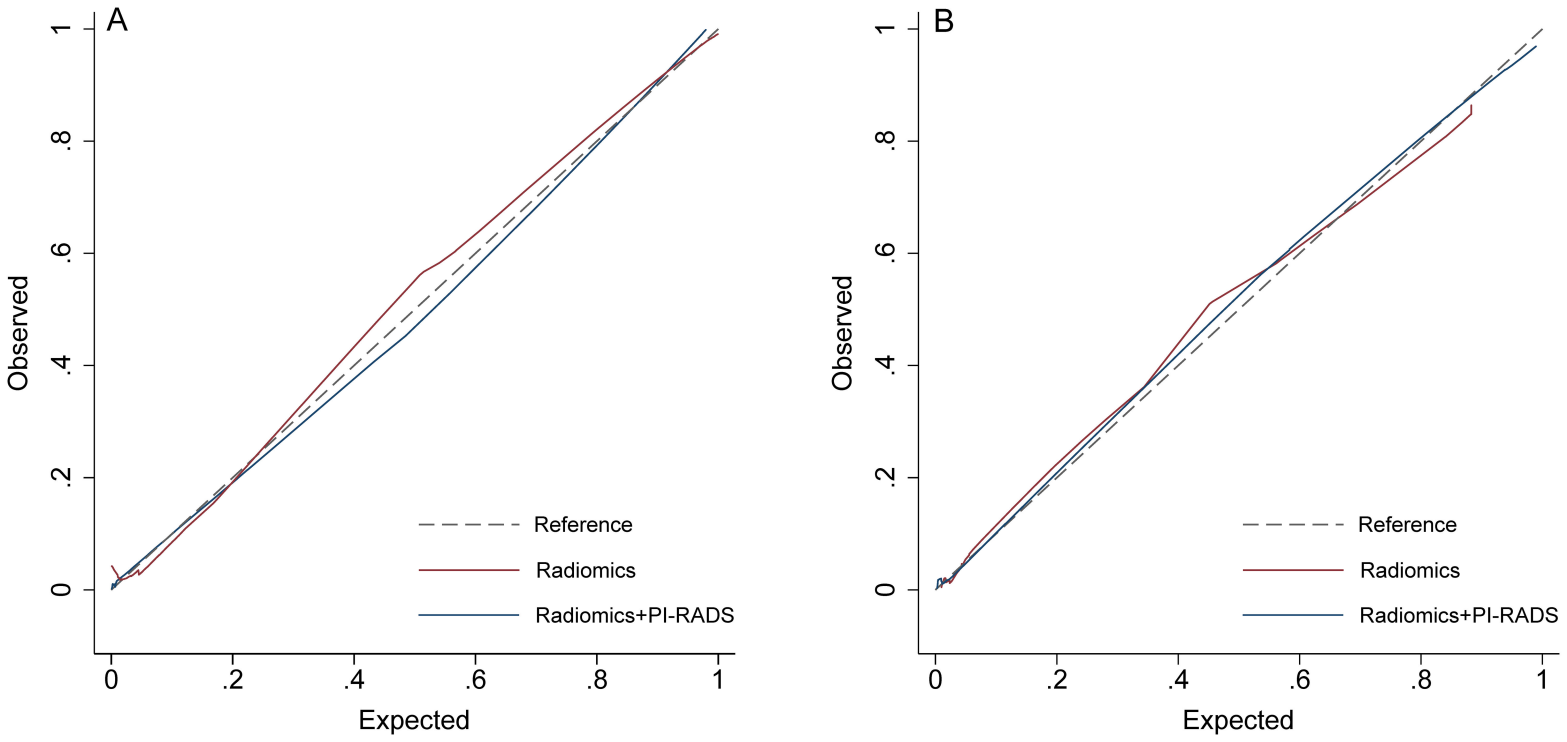
Calibration curves. **(A)** training group; **(B)** validation group. PI-RADS, Prostate Imaging Reporting and Data System, version 2.1.

On the training cohort, the radiomics model (AUC 0.943, 95% CI 0.901-0.984) performed significantly better than both PSAD (P<0.001) and PI-RADS (P<0.001), with sensitivity of 93.1% (95% CI, 83.3%-98.1%) and specificity of 88.7% (95% CI 82.3%-93.4%), respectively. When Radscore was combined with PI-RADS, diagnostic accuracy was enhanced substantially, with an AUC of 0.968 (95% CI 0.958–0.998; P=0.03). ROC curves for PSAD, PI-RADS, radiomics, and the combined model are presented in **Figure 2A** and summarized in **Table 3**. On the test cohort, radiomics also outperformed PI-RADS, with an AUC of 0.928 (95% CI 0.868–0.988) vs. 0.807 (95% CI 0.705–0.908; P=0.04). According to DeLong’s test, the combined nomogram generated higher diagnostic accuracy (AUC 0.955, 95% CI 0.905–1.00), significantly outperforming both PI-RADS (P=0.002) and radiomics alone (P=0.02), as shown in **Figure 2B**. Decision curve analysis (**Figure 3**) showed that the combined model provided superior clinical utility compared with either PI-RADS or radiomics alone. Additionally, calibration curves (**Figure 4**) confirmed excellent agreement between predicted probabilities and actual outcomes for the risk of csPCa both one the training cohort and in the test cohort.

**Table 3:** Diagnostic performance.

Indicator	Sensitivity (95% CI)	Specificity (95% CI)	LR+ (95% CI)	LR- (95% CI)	AUC (95% CI)	*P* for AUC
Training Cohort						
PSAD	44.8% (31.7%-58.5%)	85.2% (78.3%-90.6%)	3.03 (1.86-4.93)	0.65 (0.51-0.83)	0.693 (0.613-0.774)	<0.001
PI-RADS≥3	94.8% (85.6%-98.9%)	46.5% (38.1%-55.0%)	1.77 (1.5-2.09)	0.11 (0.04-0.34)	0.813 (0.752-0.873)	<0.001
PI-RADS≥4	69.0% (55.5%- 80.5%)	80.3% (72.8%-86.5%)	3.50 (2.41-5.08)	0.39 (0.26-0.57)		
Radscore	93.1% (83.3%-98.1%)	88.7% (82.3%-93.4%)	8.26 (5.18-13.20)	0.08 (0.03-0.20)	0.943 (0.901-0.984)	0.03
PI-RADS+ Radscore	94.8% (85.6%-98.9%)	88.0% (81.5%-92.9%)	7.92 (5.05-12.4)	7.92 (5.05-12.4)	0.968 (0.958-0.998)	/
Validation Cohort						
PSAD	30.4% (13.2%-52.9%)	92.1% (82.4%-97.4%)	3.83 (1.35-10.90)	0.76 (0.57-1.00)	0.722 (0.607-0.837)	0.002
PI-RADS≥3	91.3% (72.0%-98.9%)	47.6% (34.9%-60.6%)	1.74 (1.33-2.28)	0.18 (0.05-0.70)	0.807 (0.705-0.908)	0.002
PI-RADS≥4	69.6% (47.1%-86.8%)	82.54% (70.9%-90.9%)	3.98 (2.18-7.27)	0.37 (0.20-0.69)		
Radscore	91.3% (72.0%-98.9%)	87.3% (76.5%-94.4%)	7.19 (3.72-13.90)	0.10 (0.03-0.38)	0.928 (0.868-0.988)	0.02
PI-RADS+ Radscore	87.3% (66.4%-97.2%)	95.7% (86.7%-99.0%)	18.30 (5.98-55.70)	0.14 (0.05-0.39)	0.955 (0.905-1.00)	/

AUC, area under the receiver operating characteristic curve; CI, confidence interval; LR+, positive likelihood ratio; LR-, negative likelihood ratio; PI-RADS, Prostate Imaging Reporting and Data System; PPV, positive predictive value; PSAD, prostate-specific antigen density; Radscore, radiomic score.

^a^Compared with PSAD+PI-RADS.

## Discussion

In this study, we evaluated the PSAD, PI-RADS, bpMRI-based radiomics, and a combination model for identifying csPCa in men with PSA gray zone. Our analyses indicated that radiomics significantly outperformed both PI-RADS and PSAD; moreover, integrating radiomics with PI-RADS could further improve diagnostic accuracy. The consistency of results between the training and test datasets demonstrated the robustness of our findings. However, it should be noted that in this study MRI images were interpreted according to PI-RADS by two junior radiologists, which might partly explain the lower diagnostic performance compared to prior studies involving experienced readers (28). Previous studies have demonstrated the promising diagnostic value of MRI radiomics among patients suspected of PCa, with reported AUCs ranging from 0.933 to 0.941 (29–31). However, in most studies radiomic features are extracted from full mpMRI of at least 3 sequences (T2WI, DCE, ADC, and DWI) and few studies were focused on patients in PSA gray zone. In one study using bpMRI-based radiomics for csPCa, the calculated AUC was 0.844. Nevertheless, this research was focused on lesions only located in TZ, and the final model was based on TZ volume and Radscore (32).

The goal of PSA screening is to detect PCa at an early stage, and then reduce disease-specific mortality. However, relying only on PSA levels leads to a high false-positive rate, resulting in substantial over-detection of clinically insignificant, low-grade cancers (5). This may cause many unnecessary diagnostic and therapeutic interventions, as well as associated side effects, particularly in patients within the PSA gray zone. In our study, 28.57% of patients were ultimately diagnosed as benign prostatic hyperplasia or non-csPCa, suggesting many unnecessary biopsies or overtreatment. Although PI-RADS has been a consensus protocol guideline for a decade, its adoption varies widely across hospitals and institutions (33). Additionally, while mpMRI is effective for detecting PCa, it is costly and time-consuming as a screening tool. In light of this, more and more studies have explored bpMRI as an alternative to mpMRI for detecting and managing PCa, with comparable diagnostic performance while significantly reducing scan time. Nevertheless, it is reported that DCE may enhance the performance of detecting csPCa with PI-RADS scores ≥3, particularly by upgrading PI-RADS 3 lesions to PI-RADS 4 (34, 35). Conversely, Van der Leest et al. introduced a “fast bpMRI” protocol utilizing only axial T2WI for detecting csPCa in patients with PSA levels higher than 3 ng/mL (14). Their findings demonstrated that this abbreviated protocol achieved the same sensitivity as mpMRI (both 95%) with only a minor increase in false-positive rate (65% vs. 69%), although the technique requires further external validation.

In the current study, all radiomics features were extracted from two MRI protocols of T2WI and DWI. T2WI offers high soft tissue contrast and is particularly effective for delineating prostate anatomy and identifying structural abnormalities, whereas DWI provides information on tissue cellularity by assessing water molecule diffusion, which is typically restricted in cancerous tissues due to increased cellular density (36). Both T2WI and DWI indicated negative correlations with the proportion of nuclei or cytoplasm and positive correlations with luminal space percentage in prostate tissue (36). As Gleason scores increase, glandular architecture becomes more disordered, leading to a fragmented luminal appearance (17). These histopathological alterations are reflected in characteristic signal patterns on T2WI and DWI, supporting their application in radiomics-based analyses. Despite advances in auto-segmentation technology of image processing, manual delineation by radiologists is still the primary approach for lesion regions in radiomics research. However, manual segmentation remains labor-intensive and prone to variability between and within readers, which can undermine model reliability and limit clinical applicability (37). Moreover, delineating lesions on a single slice rather than across the full three-dimensional volume may fail to capture the complete morphological characteristics, reducing the accuracy of volumetric analysis. While automated segmentation offers greater efficiency, studies have reported slightly inferior performance compared to manual delineation, potentially due to the lower precision of current auto-segmentation algorithms (37). Furthermore, many automated methods are optimized for lesions in the PZ, requiring separate solutions for accurate segmentation in the TZ.

Our study has several limitations. First, the retrospective single-center study design may result in selection bias and limit the generalizability of our findings; thus, external validation is warranted. Second, MRI/US targeted biopsy results were used as the reference standard, which may lead to the omission of some MRI-invisible but pathologically significant lesions. Third, ROI delineation was performed manually by two radiologists, which may introduce potential subjectivity. Fourth, we did not assess model performance separately in the PZ and TZ due to limited subgroup sample sizes. Future research with larger and more balanced datasets is needed to evaluate diagnostic performance according to anatomical zones. Lastly, the imbalance in GS distribution between the training and test cohorts may have introduced bias in model evaluation and could potentially affect the generalizability of the model. However, it is worth noting that our dataset was split strictly with a 7:3 ratio, and the distribution of csPCa cases was also preserved in the same proportion across the cohorts.

## Conclusions

BpMRI-based radiomics significantly outperformed both PI-RADS and PSAD in predicting csPCa in men with PSA levels gray zone. Combining radiomics with PI-RADS further enhances diagnostic accuracy. Despite the promise shown by these methods, further validation and refinement, particularly in distinguishing between different anatomical zones of the prostate, are needed. This approach holds the potential to reduce unnecessary biopsies and overtreatment in those patients.

## Data Availability

The raw data supporting the conclusions of this article will be made available by the authors, without undue reservation.

## References

[1] Sung H Ferlay J Siegel RL Laversanne M Soerjomataram I Jemal A. Global cancer statistics 2020: GLOBOCAN estimates of incidence and mortality worldwide for 36 cancers in 185 countries. CA Cancer J Clin. (2021) 71:209–49. doi: 10.3322/caac.21660, PMID: 33538338

[2] Sandhu S Moore CM Chiong E Beltran H Bristow RG Williams SG. Prostate cancer. Lancet. (2021) 398:1075–90. doi: 10.1016/S0140-6736(21)00950-8, PMID: 34370973

[3] Oesterling JE. Prostate specific antigen: a critical assessment of the most useful tumor marker for adenocarcinoma of the prostate. J Urol. (1991) 145:907–23. doi: 10.1016/s0022-5347(17)38491-4, PMID: 1707989

[4] Fitzpatrick JM Banu E Oudard S. Prostate-specific antigen kinetics in localized and advanced prostate cancer. BJU Int. (2009) 103:578–87. doi: 10.1111/j.1464-410X.2009.08345.x, PMID: 19210674

[5] Pinsky PF Parnes H. Screening for prostate cancer. N Engl J Med. (2023) 388:1405–14. doi: 10.1056/NEJMcp2209151, PMID: 37043655

[6] Williams IS McVey A Perera S O’Brien JS Kostos L Chen K. Modern paradigms for prostate cancer detection and management. Med J Aust. (2022) 217:424–33. doi: 10.5694/mja2.51722, PMID: 36183329 PMC9828197

[7] Catalona WJ Smith DS Ratliff TL Dodds KM Coplen DE Yuan JJ. Measurement of prostate-specific antigen in serum as a screening test for prostate cancer. N Engl J Med. (1991) 324:1156–61. doi: 10.1056/NEJM199104253241702, PMID: 1707140

[8] Tamada T Sone T Higashi H Jo Y Yamamoto A Kanki A. Prostate cancer detection in patients with total serum prostate-specific antigen levels of 4–10 ng/mL: diagnostic efficacy of diffusion-weighted imaging, dynamic contrast-enhanced MRI, and T2-weighted imaging. AJR Am J Roentgenol. (2011) 197:664–70. doi: 10.2214/AJR.10.5923, PMID: 21862809

[9] Vilanova JC Barceló-Vidal C Comet J Boada M Barceló J Ferrer J. Usefulness of prebiopsy multifunctional and morphologic MRI combined with free-to-total prostate-specific antigen ratio in the detection of prostate cancer. Am J Roentgenol. (2011) 196:W715–22. doi: 10.2214/AJR.10.5700, PMID: 21606259

[10] Delongchamps NB Rouanne M Flam T Beuvon F Liberatore M Zerbib M. Multiparametric magnetic resonance imaging for the detection and localization of prostate cancer: combination of T2-weighted, dynamic contrast-enhanced and diffusion-weighted imaging. Bju Int. (2011) 107:1411–8. doi: 10.1111/j.1464-410X.2010.09808.x, PMID: 21044250

[11] Barentsz JO Richenberg J Clements R Choyke P Verma S Villeirs G. ESUR prostate MR guidelines 2012. Eur Radiol. (2012) 22:746–57. doi: 10.1007/s00330-011-2377-y, PMID: 22322308 PMC3297750

[12] Weinreb JC Barentsz JO Choyke PL Cornud F Haider MA Macura KJ. PI-RADS prostate imaging – reporting and data system: 2015, version 2. Eur Urol. (2016) 69:16–40. doi: 10.1016/j.eururo.2015.08.052, PMID: 26427566 PMC6467207

[13] Turkbey B Rosenkrantz AB Haider MA Padhani AR Villeirs G Macura KJ. Prostate imaging reporting and data system version 2.1: 2019 update of prostate imaging reporting and data system version 2. Eur Urol. (2019) 76:340–51. doi: 10.1016/j.eururo.2019.02.033, PMID: 30898406

[14] Udayakumar N Porter KK. How fast can we go: abbreviated prostate MR protocols. Curr Urol Rep. (2020) 21:59. doi: 10.1007/s11934-020-01008-8, PMID: 33135121

[15] Calimano-Ramirez LF Virarkar MK Hernandez M Ozdemir S Kumar S Gopireddy DR. MRI-based nomograms and radiomics in presurgical prediction of extraprostatic extension in prostate cancer: a systematic review. Abdom Radiol. (2023) 48:2379–400. doi: 10.1007/s00261-023-03924-y, PMID: 37142824

[16] Chiacchio G Castellani D Nedbal C De Stefano V Brocca C Tramanzoli P. Radiomics vs radiologist in prostate cancer. Results from a systematic review. World J Urol. (2023) 41(3):709–24. doi: 10.1007/s00345-023-04305-2, PMID: 36867239

[17] Cutaia G La Tona G Comelli A Vernuccio F Agnello F Gagliardo C. Radiomics and prostate MRI: current role and future applications. J Imaging. (2021) 7:34. doi: 10.3390/jimaging7020034, PMID: 34460633 PMC8321264

[18] Rosenkrantz AB Ginocchio LA Cornfeld D Froemming AT Gupta RT Turkbey B. Interobserver reproducibility of the PI-RADS version 2 lexicon: A multicenter study of six experienced prostate radiologists. Radiology. (2016) 280:793–804. doi: 10.1148/radiol.2016152542, PMID: 27035179 PMC5006735

[19] De Visschere P Lumen N Ost P Decaestecker K Pattyn E Villeirs G. Dynamic contrast-enhanced imaging has limited added value over T2-weighted imaging and diffusion-weighted imaging when using PI-RADSv2 for diagnosis of clinically significant prostate cancer in patients with elevated PSA. Clin Radiol. (2017) 72:23–32. doi: 10.1016/j.crad.2016.09.011, PMID: 27726850

[20] Greer MD Shih JH Lay N Barrett T Kayat Bittencourt L Borofsky S. Validation of the dominant sequence paradigm and role of dynamic contrast-enhanced imaging in PI-RADS version 2. Radiology. (2017) 285:859–69. doi: 10.1148/radiol.2017161316, PMID: 28727501 PMC5708285

[21] Obmann VC Pahwa S Tabayayong W Jiang Y O’Connor G Dastmalchian S. Diagnostic accuracy of a rapid biparametric MRI protocol for detection of histologically proven prostate cancer. Urology. (2018) 122:133–8. doi: 10.1016/j.urology.2018.08.032, PMID: 30201301 PMC6295224

[22] Niu X Chen X Chen Z Chen L Li J Peng T. Diagnostic performance of biparametric MRI for detection of prostate cancer: A systematic review and meta-analysis. Am J Roentgenol. (2018) 211:369–78. doi: 10.2214/AJR.17.18946, PMID: 29894216

[23] Bass EJ Pantovic A Connor M Gabe R Padhani AR Rockall A. A systematic review and meta-analysis of the diagnostic accuracy of biparametric prostate MRI for prostate cancer in men at risk. Prostate Cancer Prostatic Dis. (2021) 24:596–611. doi: 10.1038/s41391-020-00298-w, PMID: 33219368

[24] Cuocolo R Verde F Ponsiglione A Romeo V Petretta M Imbriaco M. Clinically significant prostate cancer detection with biparametric MRI: A systematic review and meta-analysis. Am J Roentgenol. (2021) 216:608–21. doi: 10.2214/AJR.20.23219, PMID: 33502226

[25] Alabousi M Salameh J-P Gusenbauer K Samoilov L Jafri A Yu H. Biparametric vs multiparametric prostate magnetic resonance imaging for the detection of prostate cancer in treatment-naïve patients: a diagnostic test accuracy systematic review and meta-analysis. BJU Int. (2019) 124(2):209–20. doi: 10.1111/bju.14759, PMID: 30929292

[26] DeLong ER DeLong DM Clarke-Pearson DL. Comparing the areas under two or more correlated receiver operating characteristic curves: a nonparametric approach. Biometrics. (1988) 44:837–45. doi: 10.2307/2531595 3203132

[27] Benjamini Y Hochberg Y. Controlling the false discovery rate: A practical and powerful approach to multiple testing. J R Stat Soc Ser B Methodol. (1995) 57:289–300. doi: 10.1111/j.2517-6161.1995.tb02031.x

[28] Li X Li C Chen M. Patients with “Gray zone” PSA levels: application of prostate MRI and MRS in the diagnosis of prostate cancer. J Magn Reson Imaging. (2023) 57:992–1010. doi: 10.1002/jmri.28505, PMID: 36326563

[29] Qi Y Zhang S Wei J Zhang G Lei J Yan W. Multiparametric MRI-based radiomics for prostate cancer screening with PSA in 4–10 ng/mL to reduce unnecessary biopsies. J Magn Reson Imaging JMRI. (2020) 51:1890–9. doi: 10.1002/jmri.27008, PMID: 31808980

[30] Zhang L Zhang J Tang M Lei X-Y Li L-C. MRI-based radiomics nomogram for predicting prostate cancer with gray-zone prostate-specific antigen levels to reduce unnecessary biopsies. Diagn Basel Switz. (2022) 12:3005. doi: 10.3390/diagnostics12123005, PMID: 36553012 PMC9776817

[31] Zhong J-G Shi L Liu J Cao F Ma Y-Q Zhang Y. Predicting prostate cancer in men with PSA levels of 4–10 ng/mL: MRI-based radiomics can help junior radiologists improve the diagnostic performance. Sci Rep. (2023) 13:4846. doi: 10.1038/s41598-023-31869-1, PMID: 36964192 PMC10038986

[32] Lu Y Li B Huang H Leng Q Wang Q Zhong R. Biparametric MRI-based radiomics classifiers for the detection of prostate cancer in patients with PSA serum levels of 4∼10 ng/mL. Front Oncol. (2022) 12:1020317. doi: 10.3389/fonc.2022.1020317, PMID: 36582803 PMC9793773

[33] Kim SJ Vickers AJ Hu JC. Challenges in adopting level 1 evidence for multiparametric magnetic resonance imaging as a biomarker for prostate cancer screening. JAMA Oncol. (2018) 4:1663. doi: 10.1001/jamaoncol.2018.4160, PMID: 30242308 PMC6719541

[34] Xu L Zhang G Shi B Liu Y Zou T Yan W. Comparison of biparametric and multiparametric MRI in the diagnosis of prostate cancer. Cancer Imaging. (2019) 19:90. doi: 10.1186/s40644-019-0274-9, PMID: 31864408 PMC6925429

[35] Taghipour M Ziaei A Alessandrino F Hassanzadeh E Harisinghani M Vangel M. Investigating the role of DCE-MRI, over T2 and DWI, in accurate PI-RADS v2 assessment of clinically significant peripheral zone prostate lesions as defined at radical prostatectomy. Abdom Radiol. (2019) 44:1520–7. doi: 10.1007/s00261-018-1807-6, PMID: 30361870 PMC6440804

[36] Nketiah G Elschot M Kim E Teruel JR Scheenen TW Bathen TF. T2-weighted MRI-derived textural features reflect prostate cancer aggressiveness: preliminary results. Eur Radiol. (2017) 27:3050–9. doi: 10.1007/s00330-016-4663-1, PMID: 27975146

[37] Losnegård A Reisæter LAR Halvorsen OJ Jurek J Assmus J Arnes JB. Magnetic resonance radiomics for prediction of extraprostatic extension in non-favorable intermediate- and high-risk prostate cancer patients. Acta Radiol. (2020) 61:1570–9. doi: 10.1177/0284185120905066, PMID: 32108505

